# Continuity Theory in Diphtheria—Test Abrasions

**Published:** 1879-08-20

**Authors:** A. F. Kinne

**Affiliations:** Michigan


### Southern Medical Record.

EDITORS:

T. S. POWELL, M.D. W. T. GOLDSMITH, M.D. R. C. WORD, M.D.

*R. C. WORD, M.D., Managing Editor.*

☞All Communications and Letters on Business connected with the RECORD must be addressed to the Managing Editor.

VOL. IX. ATLANTA, GA., AUGUST 20, 1879. No. 8.

### ORIGINAL AND SELECTED ARTICLES.

### *THE CONTINUITY THEORY IN DIPHTHERIA, AND THE PRACTICE OF TEST ABRASIONS.*

BY A. F. KINNE, A.M., M.D., MICHIGAN.

I was brought up to believe in the doctrine of Bretonneau—that diphtheria is a local disease—and for the most part always remains so, infecting the system, if at all, only by absorption of putrescent matters from the throat. But at length, four or five years ago, some cases came into my hands, which seemed to upset this "continuity of surface" theory and prepared me for the reception of a different one.

On the 21st of September, 1874, I was called to a farm house, some four miles out, to see L. F——, aged 17, daughter of Mr. A. F——, sick, as the messenger represented, of a "fever and sore throat."

But a little preparation was required for a trip into the country, and before we left my office, the messenger, W. F., a brother of the sick girl, aged 19 years, called my attention to a cut across the back of one of his fingers, which he had got the day before, and which, for so small an affair, was "getting to be very sore." The skin and cellular tissue had been divided by some sharp instrument down to the sheath of the extensor tendon; it was a gaping wound, and nothing had been done to bring the edges together; and the bottom of it was filled with a grayish-white exudation which reminded me of the pseudo-membranous deposits which I had often enough seen in the throat of my patients in diphtheria. I took an instrument and satisfied myself that it was adhesive, adherent to the bottom and sides of the cut, and was indeed a true diphtheritic inflammation of a clean cut, made some thirty hours before. His throat, which I proceeded at once to explore, was slightly red, but he affirmed that it was not sore and that he was well.

Educated as I had been in a different view, here was a surprise, to me at least, and possibly also a genuine discovery. For the idea that diphtheria must exist as a systemic disease in advance of the appearance of its characteristic lesions in the throat had not then, so far as I know, been demonstrated. The practice of making abrasions or scarifications upon some portion of the cutaneous surface, which this case of mine suggests, for the purpose of determining the existence of diphtheria in the system, in advance of its appearance in the fauces, and in some cases for the purpose also of making an indisputable diagnosis between diphtheria and croup, had not, at that time, so far as my reading goes, made its appearance in medical literature. And the importance of these two great objects will readily be understood and acknowledged by every practitioner. Beyond showing that both of these purposes are entirely feasible and practicable, it will not be needfull here to say another word.

Upon arrival at the farm I found, as I expected, a not severe but an unmistakable case of diphtheria; and upon repeating my visit the next day, three more cases. My messenger with the diphtheritic finger had gone on to develop the full disease; and along with him had fallen sick also two other children, a daughter, S., aged 14, and a son, C., aged 12 years. Thus I had under treatment the entire family, except the father, the mother being dead, all of whom must have taken the disease at about the same time from some common source.

With the etiology of these cases in view, I made a thorough survey of the premises, and can make my report in few words. I found the privy, the drainage of the kitchen, the cellar, the woodhouse, the cistern, the well and the entire surroundings *outside* of the house, in good order. The fault was in the house itself. It was a one-story frame building more than fifty years old; it stood flat upon the ground, and under the dining-room, sitting-room and two bed-rooms there was no cellar, and the unevenness of the floors showed that the foundations were rotten. Bear in mind that the housekeeping was in the hands of a couple of young and motherless girls, and the rest of the picture will not seem strange. Sunlight and air had been carefully excluded by heavy curtains and closed doors; the rag carpets were none too tidy, and the smell of the rooms was close, damp and musty in the extreme.

I am sorry to have to submit so meagre a report as this is upon so interesting and important a theme, for these cases suggest the point, at least, that diphtheria is a systemic disease; that it works in the blood before its local lesions make their appearance; and that it is possible, and perhaps in some cases practicable, by making abrasions or scarifications, such for instance as we use in vaccinating, to invite the disorder to the surface and gain an important advantage, in point of time, by compelling it to reveal its lurking presence in advance of its regular attack. I should be glad if I could present here some additional cases in which these views had been subjected to the test of actual experiment, but this has been impossible; diphtheria has fought shy of me and of this locality for a number of years, and I can do no better from lack of opportunity.

I must withold my paper altogether, or present it just as it is and leave it for others more favorably situated to supply its deficiencies.

In the *Virginia Medical Monthly* for September, 1876, however, there is a paper from the pen of Dr. George Bayles, one of the visiting physicians of the Northwestern dispensaries of the city of New York, some portion of which will measurably supply what we lack. It will doubtless be recollected by some that this paper was read before the New York State Medical Society, at its meeting in June, 1879, but was excluded from the printed transactions of that body, for the reason that Dr. Bayles had made use and had attributed his success, in part at least, to the use of one of Tilden & Co's proprietary preparations, their "elixir iodo-bromide of calcium compound." But with all this we need have nothing to do. The parts of the paper that subserve our purpose can be open to no sort of objection, and they amount, in brief, to this:

Dr. Bayles reports twenty cases, in all of which he was able to practice the skin abrasions as above indicated, during the incubatory stage of the disease; and in all of the cases but one he succeeded in procuring, at the seat of the test-abrasions, true traumatic patches of diptheritic exudation, in advance of the regular outset of the disease and in advance of the appearance of the characteristic pseudo-membranous deposits in the throat, by a number of hours at least.

And by a corresponding time, of course, he could be, and he was, beforehand in his treatment. There were no cases of doubtful diagnosis; he could not be otherwise than positive. Expectant remedies were therefore out of the question; he could enter at once, in advance of the usual time, upon the use of means known to be eliminative or anti-zymotic, or both perhaps.

If I may judge from my own experience or from the results of my reading, the best opening treatment in a case of diphtheria is to procure, if possible, an elimination of the poison by way of the alimentary canal. This is nothing new, however; I think I may say that this practice is a good deal in vogue upon both sides of the Atlantic. Thus the late Mr. John Scott, as reported by Dr. D. D. Howell in the

Lancet, used to treat these cases with a "scavenger," viz: of calomel, jalap and scammony, after which they usually got well. And Dr. Howell himself gives us emphatic testimony to the value of this abortive kind of treatment. Upon this side of the water I think preference is given to a cathartic dose of calomel alone; I have used myself a powder of calomel and rhubarb. Dr. Bayles reports having begun the treatment of his cases with one "courageous dose" of calomel. To this, more than to all the rest of his remedies together, coupled with the fact that this dose was given some considerable time before the disease was fully developed, is, in my opinion, to be attributed his very great success. His cases all ended in recovery; were shortened from two to four days, and were none of them followed by paralysis or other untoward results. While in the three other visiting districts, during the same year, in the care of men doubtless equally as competent as Dr. Bayles, but who employed the ordinary method of treatment, out of an aggregate of forty-seven cases there were fourteen deaths.

Can any one fail to see that we have here a matter that deserves more attention than it has hitherto received? If a patient has been exposed, or if from any other source the suspicion arises that diphtheria is impending, should we not settle the question, since we seem to have it in our power to do it so easily, and use our antidotes at the earliest possible moment? And should there in any quarter be a disposition to adopt the view of Morell McKenzie and others—that diphtheria and croup are only two different forms of one and the same disease—have we not here the means of showing easily and conclusively that his argument, though an exceedingly able one, is wholly fallacious, and his position an untenable one?

